# Dural tear with severe irrigation-related complications during unilateral biportal endoscopy under general anesthesia: a case series and literature review

**DOI:** 10.3389/fmed.2026.1776670

**Published:** 2026-02-24

**Authors:** Jian Guo, Feng Zhou, Yitao Qian, Yuting Qiu, Shuangjian Han, Jianhong Xu

**Affiliations:** Department of Anesthesiology, The Fourth Affiliated Hospital of School of Medicine, International School of Medicine, International Institutes of Medicine, Zhejiang University, Yiwu, China

**Keywords:** dural tear, emergence agitation, hypertension, irrigation-related complications, tachycardia, unilateral biportal endoscopy

## Abstract

**Background:**

Unilateral biportal endoscopy (UBE) has been widely adopted in clinical practice owing to its advantages of providing a clearer surgical field, reducing estimated blood loss, and shortening hospitalization duration. Dural tear represents a common complication of UBE; when combined with the unique dual-channel continuous high-pressure irrigation system, it may trigger severe irrigation-related complications (IRC) that jeopardize patient safety.

**Case presentation:**

We retrospectively reviewed UBE procedures performed at the Fourth Affiliated Hospital of School of Medicine Zhejiang University from August 2024 to July 2025. A total of 5 cases of severe IRC following incidental dural tear of UBE were identified. Key clinical manifestations during the anesthesia emergence phase included refractory hypertension, tachycardia, postoperative emergence agitation, headache, and back pain. All patients achieved successful outcomes following comprehensive treatment.

**Conclusion:**

Incidental dural tear during UBE can result in severe IRC, which pose a considerable threat to patient safety. Comprehensive interventions—including sedation, analgesia, targeted management of hypertension and tachycardia, as well as administration of mannitol, furosemide, or methylprednisolone—are crucial. Anesthesiologists should maintain vigilance for these clinical features and proactively manage IRC associated with dural tear during UBE.

## Background

Since 2017, the unilateral biportal endoscopy (UBE) has been increasingly adopted for the minimally invasive treatment of spinal disorders, such as lumbar spinal stenosis, lumbar disk herniation, lumbar foraminal stenosis, lumbar intraspinal synovial cysts, epidural lipomatosis, lumbar spondylolisthesis, intervertebral space infection, and revision surgery (1, 2). For single-level discectomy, UBE achieves clinical outcomes comparable to open lumbar microdiscectomy—including pain control, functional recovery, and patient satisfaction—while offering advantages such as minimal blood loss, shorter hospital stays, and reduced postoperative back pain (3). Yu et al. (4) confirmed that UBE’s dual independent yet interconnected channels integrate the benefits of microscopic surgery and interlaminar endoscopy, enhancing procedural flexibility, accuracy, and reliability, which might provide a broad and clear surgical field, minimizes tissue damage, and accelerates patient recovery.

With the growing volume of UBE procedures, associated complications have been reported, including epidural hematoma, dural sac tear, retroperitoneal effusion, inadequate decompression, postoperative back pain/headache, early recurrence, iatrogenic spinal instability, anemia, and infection, which can prolong hospitalization and significantly impact patient satisfaction (4, 5).

Incidental dural tear is a common complication of the UBE, ranging in incidence from 0.9 to 13.2%, which can lead to cerebrospinal fluid leakage, pseudomeningocele, infection, or meningitis if not treated properly (1, 6). Notably, rare but challenging irrigation-related complications (IRC) can occur during or after surgery, posing dilemmas for perioperative management (2).

Based on all clinical data, including the entire process of anesthesia and surgery, as well as the patient’s clinical manifestations and outcomes, we identified a total of 5 cases of severe IRC following incidental dural tear of UBE between August 2024 and July 2025, without exclusion. These cases presented with unique clinical manifestations—including unexplained refractory hypertension, tachycardia, postoperative emergence agitation, headache, and back pain—that endangered patient safety. These cases highlight the need for anesthesiologists to recognize and promptly manage such complications.

## Case presentation

Five cases of severe IRC following incidental dural tear of UBE were identified performed at the Fourth Affiliated Hospital of Zhejiang University School of Medicine between August 2024 and July 2025. The study was approved by the hospital’s Ethics Committee (Approval No.: K2025281), and written informed consent for publication was obtained from all patients or their legal representatives. The case series was reported in accordance with CARE guidelines. Baseline characteristics of the five patients are summarized in Table 1.

**Table 1:** Basic information of five patients.

Case	1	2	3	4	5
Age (Y)	51	75	63	70	35
Gender (M/F)	M	F	F	F	M
Height (cm)	170	150	158	150	167
Weight (Kg)	68	45	52	55	82
BMI	23.53	20.00	20.83	24.44	29.40
Comorbidity	NO	Hypertension, stroke	No	Hypertension	Hypertension
History of surgery (Y/N)	Y	Y	Y	Y	N
ASA classification	I	II	I	II	II
Diagnosis	1. Lumbar disk herniation with sciatica (L5/S1) 2. Lumbar canal stenosis (L5/S1)	1. Lumbar disk herniation (L4/5) 2. Lumbar spinal stenosis (L4/5)	Lumbar disk herniation with sciatica (L4/5)	Lumbar disk herniation (L4/5)	Lumbar disk herniation with sciatica (L5/S1)

BMI, body mass index; ASA, American Society of Anaesthesiologists.

### Case 1

A 51-year-old male with lumbar disk herniation and lumbar spinal stenosis (L5/S1) underwent UBE-assisted spinal canal decompression, discectomy, and nerve release. Intraoperatively, a dural tear was identified, but hemodynamic and respiratory parameters remained stable. The surgery lasted 93 min without significant circulatory fluctuations.

Postoperatively, the patient was transferred to the Post-anesthesia care unit (PACU) for 165 min. Upon awakening, he developed refractory hypertension (blood pressure [BP]: 215/111 mmHg), sinus tachycardia (heart rate [HR]: 144 bpm), headache, and back discomfort. Spinal surgeons confirmed severe IRC. Intermittent doses of urapidil and nicardipine yielded unsatisfactory results.

Clinical symptoms improved after administration of hydrocodone hydrochloride (5 mg), furosemide (20 mg), and methylprednisolone (40 mg), and the patient was transferred to a general ward.

### Case 2

A 75-year-old female patient with a history of hypertension and stroke was diagnosed with lumbar disk herniation and lumbar spinal stenosis (L4/5). She underwent UBE-assisted spinal canal decompression and discectomy.

The surgery lasted 250 min, with an estimated blood loss of 100 mL and urine output of 950 mL. After tracheal extubation, the patient developed refractory hypertension (BP: 203/95 mmHg), sinus tachycardia (HR: 132 bpm), and severe emergence agitation (Richmond Agitation-Sedation Scale score: +4).

Elevation of propofol (50 mg, intravenous bolus), sufentanil (10 μg, intravenous bolus), and intermittent doses of urapidil (10 mg each, total 30 mg) and esmolol (20 mg each, total 60 mg) resulted in modest improvements in blood pressure and heart rate. Clinical symptoms significantly resolved after administration of mannitol (250 mL, intravenous infusion over 30 min). The patient was transferred to a ward after a 100-min PACU stay.

### Case 3

A 63-year-old female underwent UBE-assisted spinal canal decompression and discectomy for lumbar disk herniation (L4/5). The surgery lasted 144 min, with an estimated blood loss of 80 mL and urine output of 900 mL. Also, incidental dural tear occurred during the surgery.

During the recovery phase, the patient developed refractory hypertension (BP: 205/103 mmHg), sinus tachycardia (HR: 151 bpm), moderate emergence agitation (Richmond Agitation-Sedation Scale score: +3), and severe muscle rigidity (modified Ashworth Scale score: 4).

Sedation with propofol (50 mg, intravenous bolus), analgesia with sufentanil (10 μg, intravenous bolus), dehydration with furosemide (40 mg, intravenous bolus), and anti-inflammatory therapy with methylprednisolone (80 mg, intravenous bolus) led to minimal improvement. She subsequently developed severe electrolyte disturbances and refractory ventricular premature contractions. The patient was transferred to the Intensive Care Unit (ICU) after 90 min of symptomatic treatment in the PACU.

### Case 4

A 70-year-old female with lumbar disk herniation (L4/5) underwent UBE-assisted spinal canal decompression and discectomy. After extubation, the patient developed hypertension (BP: 186/90 mmHg), sinus tachycardia (HR: 147 bpm), severe back pain (Numerical Rating Scale score: 8/10), and mild agitation (Richmond Agitation-Sedation Scale score: +2). Sedation with propofol (30 mg, intravenous bolus), analgesia with sufentanil (5 μg, intravenous bolus), administration of furosemide (20 mg, intravenous bolus), and urapidil (12.5 mg, intravenous bolus) significantly stabilized hemodynamics (BP: 152/85 mmHg; HR: 118 bpm). However, neurological examination revealed apathy and reduced adherence to instructions. The patient was transferred to the ICU for further management.

### Case 5

A 35-year-old male with a history of hypertension (uncontrolled, no regular medication) underwent UBE-assisted spinal canal decompression and discectomy for lumbar disk herniation (L5/S1). Upon awakening, he developed severe hypertension (BP: 227/132 mmHg), sinus tachycardia (HR: 143 bpm), and refractory agitation (Richmond Agitation-Sedation Scale score: +5, requiring physical restraint).

Treatment with propofol (50 mg, intravenous bolus), methylprednisolone (40 mg, intravenous bolus), and intermittent doses of urapidil (10 mg each, total 40 mg) and esmolol (20 mg each, total 60 mg) resulted in significant improvement (BP: 145/82 mmHg; HR: 105 bpm; Richmond Agitation-Sedation Scale score: 0). The patient was transferred to a ward after a 115-min PACU stay.

Perioperative details, clinical manifestations, treatments, and outcomes are summarized in Tables 2–4, respectively.

**Table 2:** Perioperative related information.

Case	1	2	3	4	5
Operation time (min)	93	250	144	107	111
Anesthesia duration (min)	130	280	215	165	145
Intraoperative use of vasoactive drugs (Y/N)	N	Y	Y	Y	Y
Blood Loss (mL)	30	100	30	30	20
Urine Output (mL)	250	950	900	300	200
Dural Tear (Y/N)	Y	Y	Y	Y	Y

**Table 3:** Clinical manifestations and treatments.

Case	1	2	3	4	5
The highest BP (mmHg)	215/111	203/95	205/103	186/90	227/132
The highest HR (bpm)	144	132	151	147	143
Headache (Y/N)	Y	N	N	N	N
Back pain (Y/N)	Y	N	N	Y	N
Postoperative agitation (Y/N)	N	Y	Y	Y	Y
High muscle tension (Y/N)	N	N	Y	N	N
Sedation (Y/N)	N	Y	N	Y	Y
Analgesia (Y/N)	N	N	Y	Y	N
Furosemide (Y/N)	Y	N	Y	Y	N
Mannitol (Y/N)	N	Y	N	N	N
Hormone (Y/N)	Y	N	Y	N	Y
antihypertensive drugs (Y/N)	Y	Y	Y	Y	Y

ICU, intensive care unit.

**Table 4:** Prognosis and outcome.

Case	1	2	3	4	5
PACU dwell time (min)	165	100	90	115	115
Postoperative destination	Ward	Ward	ICU	ICU	Ward
Length of hospital stay (D)	12	21	7	11	10

PACU, post-anesthesia care unit.

## Discussion

We report 5 cases of severe IRC following incidental dural tear of UBE. Clinical manifestations, primarily refractory hypertension, tachycardia, emergence agitation, headache, back pain, and muscle rigidity, emerged during the postoperative anesthesia recovery phase. These life-threatening complications warrant close attention from anesthesiologists. A comprehensive management approach is required for such complications, encompassing dehydration therapy, steroid administration, antihypertensive intervention, analgesia, and sedation.

### Historical context of UBE

De Antoni et al. (7) first modified a technique to access the epidural space via a direct posterior portal with dual channels on the same side. Not until 2013, when Soliman (8) reported satisfactory outcomes of dual-channel irrigation endoscopic discectomy for lumbar disk herniation, did this technique garner widespread attention. Korean scholars formally coined the term “unilateral biportal endoscopy (UBE)” in 2016, defining it as a technique requiring separate working and viewing channels on one side with continuous positive-pressure irrigation (20–30 mmHg) (9, 10).

Unlike traditional percutaneous endoscopic spine surgery, which is limited by single-cannula operation in terms of visualization, maneuverability, and efficiency, UBE offers a superior surgical field, greater operational flexibility, a shorter learning curve, more accessible instruments, and reduced intraoperative fluoroscopy radiation (11, 12). The technique has been widely adopted in regions such as the United Arab Emirates, Egypt, and China (13), and its promotion in recent years is attributed to benefits including shorter hospital stays, lower blood loss, and comparable complication rates (14, 15).

### Mechanisms of IRC following dural tear

As previously noted, incidental dural tear is a common complication of UBE (1). Lewandrowski et al. (16) reviewed 64,470 endoscopic procedures and reported an overall incidental dural tear incidence of 1.07%. Contributing factors include surgical-related issues (e.g., surgeon inexperience with endoscopic anatomy, inadequate hemostasis leading to obscured visualization) and patient-related factors (e.g., female gender, age > 70 years, severe lumbar spinal stenosis, lumbar spondylolisthesis, articular facet cysts) (15, 17–20).

Management of incidental dural tear typically depends on the size of the dural sac injury: small tears (<5 mm) are often managed with fibrin sealant or close observation, while larger tears (>5 mm) require suturing (9, 21). In our 5 cases, surgeons implemented appropriate interventions for dural tear, yet severe IRC still occurred. These complications are linked to UBE’s unique pressure pump irrigation system, which can trigger a spinal cord hypertension response that threatens patient life (2). Some Chinese scholars refer to this phenomenon as “myeloid hypertension-like syndrome,” which is linked to elevated cerebrospinal fluid pressure and spinal cord oedema (22).

Previous literature reports have shown that when incidental dural tear occurs, surgeons tend to focus more on nerve damage or cerebrospinal fluid leakage, while overlooking IRC (16). All 5 patients presented with relevant clinical manifestations after surgery, which may be related to two factors. First, the sedative and analgesic effects of anesthesia during surgery mask the corresponding clinical manifestations. Second, dural tear combined with continuous pressure pump irrigation resulted in delayed spinal cord hypertension, spinal cord oedema, and corresponding clinical manifestations. Actually, once dural tear occurs during the UBE, anesthesiologists need to be alert to the occurrence of severe IRC.

### Clinical manifestations and management rationale

All 5 patients developed refractory hypertension and tachycardia during postoperative recovery, possibly attributable to three mechanisms: (1) Continuous high irrigation pressure during UBE may increase epidural and intracranial pressure, triggering the Cushing reflex (hypertension, tachycardia, bradycardia in severe cases) (23); (2) Incidental dural tear and retrograde irrigation may elevate intradural pressure, which is transmitted to the cervical spine and intracranial cavity, inducing sympathetic hyperactivity; (3) Inadequate postoperative pain control (e.g., back pain and headache) may activate the sympathetic nervous system, contributing to hypertension and tachycardia.

Additionally, 4 of the 5 patients experienced varying degrees of emergence agitation—a condition associated with multiple factors, including patient characteristics (e.g., age > 65 years, preoperative anxiety), disease status (e.g., spinal cord irritation), and anesthesia method (e.g., use of volatile anesthetics) (24, 25). Severe IRC may also precipitate emergence agitation by increasing spinal cord irritation, which requires prompt sedation and analgesia.

Among the 5 patients, 1 experienced headache and 2 reported back pain. Postoperative low back pain and headache are common complications of UBE, associated with increased intradural and intracranial pressure from irrigation fluid (1, 8, 26). Notably, these symptoms may be exacerbated in the presence of incidental dural tear, as irrigation fluid directly irritates the spinal cord and meninges.

### Prevention and management strategies

Prevention and management of severe IRC following incidental dural tear during UBE require a multimodal approach:

1. Intraoperative prevention: Strictly adhere to surgical indications (e.g., avoid UBE in patients with severe dural adhesion), shorten surgical duration (<180 min to reduce irrigation volume), maintain irrigation pressure < 25 mmHg (to avoid excessive intraspinal pressure), and use isotonic saline as irrigation fluid (to prevent electrolyte disturbances) (8, 27). Intraspinal pressure monitoring may help detect severe IRC following incidental dural tear early and facilitate timely treatment (28).
2. Postoperative monitoring: Closely monitor vital signs (BP, HR) and neurological status (level of consciousness, muscle tone) for 30–60 min after awakening; promptly identify refractory hypertension (>180/110 mmHg) or tachycardia (>130 bpm).
3. Targeted treatment: Comprehensive treatment—including dehydration, steroid therapy, antihypertensive intervention, sedation and analgesia—is critical. Additionally, multimodal analgesia during the perioperative period of spinal surgery is essential, as adequate pain management correlates with reduced emergence agitation and improved patient outcomes (29).

## Conclusion

Incidental dural tear during UBE can lead to severe IRC, which pose a significant threat to patient safety. Clinical manifestations typically include unexplained refractory hypertension, tachycardia, postoperative emergence agitation, headache, and back pain during the anesthesia awakening phase.

Comprehensive treatments—including sedation, analgesia, antihypertensive therapy, and administration of mannitol, furosemide, or methylprednisolone—are critical. Anesthesiologists should remain vigilant for these clinical signs, collaborate closely with spinal surgeons to monitor intraoperative dural integrity, and implement proactive management strategies to improve patient outcomes.

Future studies with larger sample sizes are needed to clarify the incidence and risk factors of IRC following incidental dural tear of UBE, and to develop standardized prevention and treatment protocols.

## Data Availability

The original contributions presented in the study are included in the article/supplementary material, further inquiries can be directed to the corresponding author.
